# Can high COVID-19 vaccination rates in adults help protect unvaccinated children? Evidence from a unique mass vaccination campaign, Schwaz/Austria, March 2021

**DOI:** 10.2807/1560-7917.ES.2022.27.39.2101027

**Published:** 2022-09-29

**Authors:** Hannes Winner, Janine Kimpel, Florian Krammer, Dorothee von Laer, Jörg Paetzold

**Affiliations:** 1 University of Salzburg, Department of Economics, Salzburg, Austria; 2 Institute of Virology, Department of Hygiene, Microbiology and Public Health, Medical University of Innsbruck, Innsbruck, Austria; 3 Department of Microbiology, Icahn School of Medicine at Mount Sinai, New York, United States

**Keywords:** COVID-19, mass vaccination, indirect protective effect, population immunity

## Abstract

**Background:**

After an outbreak of the SARS-CoV-2 Beta variant in the district of Schwaz/Austria, vaccination with Comirnaty vaccine (BNT162b2 mRNA, BioNTech-Pfizer) had been offered to all adult inhabitants (≥ 16 years) in March 2021. This made Schwaz one of the most vaccinated regions in Europe at that time (70% of the adult population took up the offer). In contrast, all other Austrian districts remained with low vaccine coverage.

**Aim:**

We studied whether this rapid mass vaccination campaign provided indirect protection to unvaccinated individuals such as children (< 16 years) living in the same district.

**Methods:**

To study the effect of the campaign we used two complementary approaches. We compared infection rates among the population of children (< 16 years) in Schwaz with (i) the child population from similar districts (using the synthetic control method), and (ii) with the child population from municipalities along the border of Schwaz not included in the campaign (using an event study approach).

**Results:**

Before the campaign, we observed very similar infection spread across the cohort of children in Schwaz and the control regions. After the campaign, we found a significant reduction of new cases among children of −64.5% (95%-CI: −82.0 to −30.2%) relative to adjacent border municipalities (using the event study model). Employing the synthetic control method, we observed a significant reduction of −42.8% in the same cohort.

**Conclusion:**

Our results constitute novel evidence of an indirect protection effect from a group of vaccinated individuals to an unvaccinated group.

**Public health impact of this article**

**What did you want to address in this study?**

Current COVID-19 vaccines have a high efficacy in preventing symptomatic infections, especially for pre-Omicron variants, and severe disease for all variants. We investigated whether a mass vaccination campaign conducted in the adult population in the district Schwaz/Austria in March 2021 had an indirect protective effect on unvaccinated children.

**What have we learnt from this study?**

SARS-CoV-2 infections were reduced in the unvaccinated children in the district Schwaz/Austria following the mass vaccination compared with border municipalities in the neighbouring district or with a control region.

**What are the implications of your findings for public health?**

Our results demonstrate that high COVID-19 vaccine coverage in the population can provide indirect protection for groups where a vaccine is not yet approved or vaccine-induced immunity may be poor (e.g. old age or underlying conditions).

## Introduction

In 2021, many countries still did not have vaccines against coronavirus disease (COVID-19) available for young age cohorts. In addition, some parents were and still are hesitant regarding potential risks and benefits of inoculating their children, meaning that vaccination coverage for this population remains modest [1-4]. This raises the important question whether population immunity can be achieved by high vaccination rates when a sufficiently large share of vaccinated adults provide indirect protection to unvaccinated individuals in the community [5,6]. If this indirect vaccination effect exists, a high coverage among older cohorts may protect younger cohorts such as children from infection [7]. More generally, community protection may help contain the pandemic even in the presence of groups unwilling or unable to get vaccinated.

To analyse this indirect protection effect, we studied a unique rapid mass vaccination campaign. In particular, following an outbreak of the Beta variant (Phylogenetic Assignment of Named Global Outbreak (Pango) lineage designation B.1.351) of severe acute respiratory syndrome coronavirus 2 (SARS-CoV-2) in the district of Schwaz (Austria), the government of Austria supplied 100,000 extra doses of the Comirnaty vaccine (BNT162b2 mRNA, Pfizer/BioNTech) to rapidly mass-vaccinate the entire adult population (≥ 16 years) of Schwaz [8,9]. After the first campaign weekend in March 2021, around 70% of the adult population of Schwaz (71,463 people) had received their first dose. In contrast, the rest of the country had a very low vaccination coverage (first dose) of around 10% at that time [8,9]. This local mass vaccination campaign created a situation in which the vaccination coverage of the adult population differed sharply at the district border of Schwaz, while the coverage of those younger than 16 years remained the exact same, basically zero (the European Medicines Agency approved the first vaccine for those under 16 years only on 28 May 2021). We exploited this sharp difference in adult vaccination rates to study the indirect protection effect on unvaccinated children. It is important to note that the SARS-CoV-2 Alpha variant (B.1.1.7) was the dominant variant in Austria at the time of our study [10].

## Methods

### Study design and data sources

Our study design compared unvaccinated age cohorts (younger than 16 years) in the district of Schwaz with the same age cohort in the control regions before and after the mass vaccination campaign. The study population comprised of two age cohorts: The first age cohort were children under the age of 16 years, who remained unvaccinated. The second cohort were individuals aged 16–50 years, who are likely to represent the population that interacts the most with the cohort of children under 16 years of age [11]. As outcome variable we employed all infections by age group recorded in Schwaz and the control regions. We used data from the Austrian epidemiological reporting system (Österreichisches Epidemiologisches Meldesystem (EMS)). These data comprise epidemiological data at municipality and district-level from all Austrian districts and the municipalities within those districts.

Our research design rested on two alternative approaches to estimate the effect of the campaign: Firstly, we apply the *synthetic control method*, which compared the district of Schwaz with a control group of highly similar districts. Secondly, we compared infection dynamics in municipalities along the district border of Schwaz using an *event study (difference-in-difference (DID))* design. This research design has already been used in a related study analysing the effect of the mass vaccination campaign on infection rates of the adult population [9]. For more methodological details we refer to that study.

### Schwaz vs synthetic control group

The synthetic control method is widely applied in causal analysis [12] and also in recent health and COVID-19 research [13,14]. Using this method, we selected from all 91 Austrian districts the districts which approximated as closely as possible the pre-intervention characteristics of Schwaz. The selection of this synthetic control group was based on a number of variables, namely the spread of SARS-CoV-2 infection before the vaccination campaign as well as the population size, geographical area size and number of municipalities within a district. Supplementary Table S1 provides descriptive statistics of Schwaz and the chosen control regions. Infection rates prior to the mass vaccination campaign were comparable in Schwaz and the control group as we previously published [9]. We then compared age group-specific incidence rates between Schwaz and its synthetic counterpart before and after the mass vaccination campaign. We also executed a placebo in-space exercise to draw inferences and, following [15], provided confidence sets for the estimated cumulative effects of the vaccination campaign (details on pre-treatment profiles of Schwaz and the synthetic control group are provided in Supplementary Table S1; the results of the placebo in-space test are shown in Supplementary Figure S1).

### Schwaz vs border municipalities

As a second approach, we exploited the fine geographical variation the mass vaccination campaign created and compared Schwaz with bordering municipalities that were not included in the campaign. For this analysis we used an event-study model based on a DID design to estimate the effect of the campaign on the incidence among the people younger than 16 years in Schwaz relative to the same age group in the border municipalities [16]. Supplementary Table S2 provides municipality-level descriptive statistics of Schwaz and the bordering municipalities included in the study, showing great similarity between the two groups regarding geographical and sociodemographic characteristics. The dependent variable was the number of new infections per 100,000 inhabitants for the age group younger than 16 years. We executed a two-way fixed effects model with an indicator variable for municipalities from the district of Schwaz. We calculated for each week *k* the DID in the 7-day moving average of new infections per 100,000 inhabitants for children younger than 16 years in the bordering municipalities and Schwaz. The regression equation is given by


$$y_{it,w}=\delta_{i}+\delta_{w}+{\sum_{k=-6}^{-1}\beta_{k}D_{it,w}+\sum_{k=1}^{11}\beta_{k}D_{it,w}+\epsilon }_{it,w}\;\;(Formula\;1),$$

Where *y_it,w_* denotes the 7-day moving average of new infections per 100,000 for children below 16 years from municipality i (Schwaz or border municipalities) on day *t,* which is nested in week *w*. *δ_i_* and *δ_w_* represent municipality- and week-fixed effects, respectively. *D_it,w_* is a binary variable taking a value of 1 for municipalities in Schwaz and 0 for border municipalities just outside of Schwaz. *k* in the sum operators stands for the weeks before (first sum) and after (second sum) the vaccination campaign. *β_k_* depicts the difference in outcomes (e.g. incidence among children younger than 16 years) between Schwaz and the border municipalities in any given week relative to the week when the first dose of the vaccination campaign was given (calendar week 10) [17].

## Results

### Impact of the mass vaccination campaign on vaccine coverage

**Figure 1:**
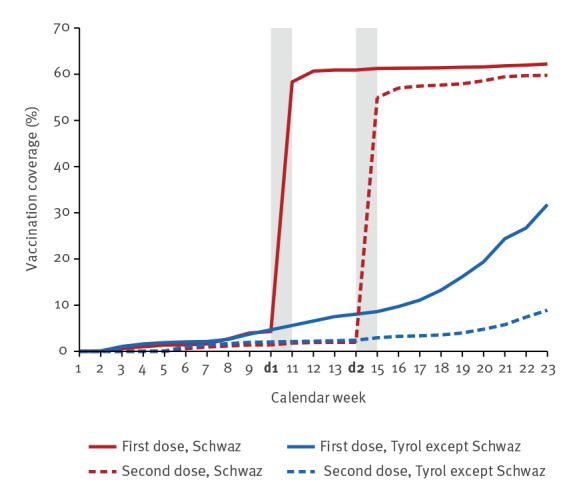
COVID-19 vaccination coverage in people aged 16–50 years in Schwaz vs the rest of Tyrol, Austria, March 2021

To illustrate the stark difference in vaccine coverage after the mass vaccination campaign we display the shares of individuals aged 16–50 years who received the first and second dose. Figure 1 plots vaccination rates of this age group for the district of Schwaz vs all other Tyrolian districts (pooled together). The impact of the mass vaccination campaign in Schwaz vis-à-vis the other districts was striking. Before the first dose of the campaign (11–16 March), vaccination coverage (one dose) among the 16–50-year-olds was exactly the same between Schwaz and everywhere else, at around 5%. After the campaign, this vaccination coverage (one dose) increased more than 10-fold in Schwaz, to around 60%. In contrast, for children below 16 years, vaccines were not available (except off-label), and vaccination rates among this population remained the same between Schwaz and the control regions (see Supplementary Figure S2 for the vaccination coverage in children).

COVID-19: coronavirus disease.

The shaded areas indicate the roll-out periods of the mass vaccination (d1: 11–16 of March 2021, calendar week 10, and d2: 8–11 April 2021, calendar week 14).

### Impact of the vaccination campaign on SARS-CoV-2 infections in children: Schwaz vs synthetic control group

**Figure 2:**
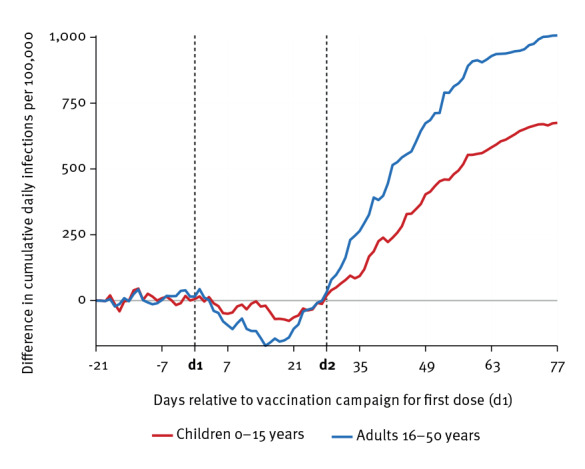
Difference in cumulative daily SARS-CoV-2 infections by age group between the synthetic control group and Schwaz, Austria, March 2021

D1: first dose; d2: second dose; SARS-CoV-2: severe acute respiratory syndrome coronavirus 2.

Figure 2 depicts the difference in cumulative daily infections by age group between the synthetic control region and Schwaz (sample size for children in Schwaz/control group: 12,993/13,337; and for adults: 37,652/19,851; see Supplementary Table S1 for further details). The age groups from Schwaz and the control region had very similar levels of SARS-CoV-2 infections before the first dose of the mass vaccination campaign. While shortly after the first dose, the control region had somewhat lower daily infections, infection levels diverged with the second dose of the campaign: At the end of the observational period (28 May), we observed a difference of 1,007.2 (95% confidence interval (CI): 416.1–1,627.1) cumulative daily infections per 100,000 inhabitants for the adults aged 16–50 years (2,762.8 cumulative daily infections per 100,000 in the control group vs 1,755.6 in Schwaz). This figure translates into a difference of 57.4%. For children below 16 years, we found a difference of 675.3 avoided infections per 100,000 (95% CI: 146.9–1,232.6; 2,253.1/100,000 in the control group vs 1,577.8/100,000 in Schwaz), a relative difference of 42.8%.

A positive difference in cumulative daily infections indicates higher infection rates in the control region than for Schwaz. The pre-campaign period is defined as 21 days before the first dose, the post-campaign period ended 77 days after the first dose (i.e. 28 May). The vertical dashed lines represent the first dose (d1) and the second dose (d2) administered as part of the mass vaccination campaign. The horizontal line denotes a zero difference in cumulative daily infections between Schwaz and the control region.

### Impact of the vaccination campaign on SARS-CoV-2 infections in children: Schwaz vs border municipalities

In a second approach, we compared Schwaz with border municipalities that were not included in the campaign. 

**Figure 3:**
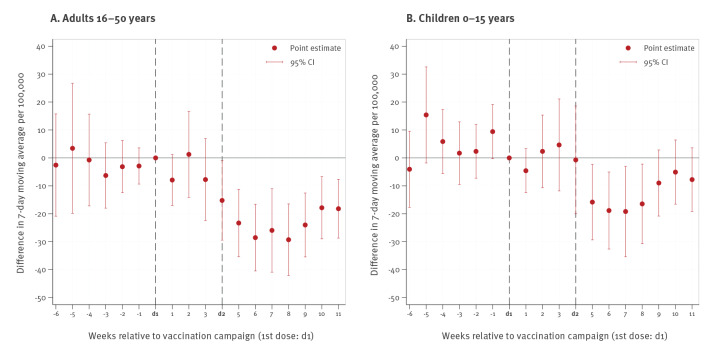
Daily infections with SARS-CoV-2 in the two age groups, Schwaz, Austria, March 2021

CI: confidence interval; d1: first dose; d2: second dose; SARS-CoV-2: severe acute respiratory syndrome coronavirus 2.

Figure 3 plots the weekly campaign effect of an event-study model according to Formula 1, displaying the difference between Schwaz and the border municipalities relative to the week when the first dose of the campaign took place, calendar week 10-2021). In the weeks before the mass vaccination campaign, we did not find any statistically significant difference in infection levels between Schwaz and the border municipalities. After the second dose from the mass vaccination campaign, the number of new cases in Schwaz for both age groups decreased significantly vis-à-vis the border municipalities. Furthermore, we used a standard two-period (before/after) DID regression to estimate the average reduction in daily infections across all post-campaign weeks (Figure 3) [9]. We found a significant reduction of new cases after the campaign (relative to the border municipalities) of −75.1% (95% CI: −85.8 to −47.8%) for adults aged 16–50 years. For children below 16 years, we observed a significant reduction of −64.5% (95% CI: −82.0 to −30.2%).

The figure displays the results from regression Formula 1 and uses the 7-day moving average of daily cases per 100,000 as outcome variable for Schwaz and its bordering municipalities. The plotted coefficients represent the weekly difference in the 7-day moving average of new cases between Schwaz and the border municipalities relative to the reference period (week when dose 1 of campaign was administered, i.e. calendar week 10 of 2021). The vertical dashed lines represent the first dose (d1) and the second dose (d2) administered as part of the mass vaccination campaign.

## Discussion

In this study, we scrutinised the impact of community-level protection on incidence rates of unvaccinated children. We exploited a unique rapid mass vaccination campaign to estimate this indirect protection effect on unvaccinated children under the age of 16 years. As controls we used the same age cohorts from comparable but untreated districts and border municipalities (i.e. without community-level protection) which followed very similar trends in infection spread before the campaign.

We first documented considerable vaccine uptake that raised coverage from 5% to 60% for the population between 16 and 50 years old through the campaign, which is broadly in line with earlier evidence on the vaccination campaign in Schwaz [9]. More importantly, our analysis showed that the substantial rollout of the Comirnaty vaccine in Schwaz was also accompanied by a significant reduction of 40–65% in new SARS-CoV-2 infections in the age cohort of unvaccinated children relative to the same age cohort in the control regions. This constitutes a systematic and substantial indirect protection effect from vaccinating a majority of the adult population.

So far, evidence of this indirect protection effect from mass vaccination against COVID-19 is scarce. To the best of our knowledge, only one study estimated this indirect effect of population-wide mass vaccination coverage [10], focusing on the community-level temporal variation in vaccine coverage in Israel and relating this coverage to the temporal variation of positive SARS-CoV-2 tests. The underlying variation in vaccine coverage between the two time periods that study employed was modest, ranging from a 5 to a maximum 20 percentage point change in the fraction of vaccinated individuals. In contrast, the variation in vaccination coverage in our study was considerably larger, with coverage jumping from around 10% to more than 70% within one weekend. Thus, our analysis is very well suited to study the potential effect of community-level protection. Our results demonstrating indirect protection are further supported by studies showing a lower secondary attack rate in unvaccinated household members when the index case was fully vaccinated compared with an unvaccinated index case [18,19].

Our study has potential limitations. Firstly, our study was not a randomised clinical trial but an observational study, which may be influenced by confounders such as lockdown policies. Although most non-pharmaceutical interventions (such as school closures or mask mandates) were identical for Schwaz and the control regions, there was an additional requirement to take SARS-CoV-2 test between 11 March and 8 April when crossing the district border [9]. This test requirement may have affected the spread of infections. However, we previously showed that in none of the five other Austrian districts that had the same test requirement, did infection numbers drop at a similar magnitude as they did in Schwaz after the campaign [9].

Secondly, while our DID design controlled for time-varying general trends over time in infection spread (such as a third wave), we could not account for changing individual behaviour such as vaccinated individuals being less mindful of physical distancing measures. However, a previous analysis of mobility data did not show large differences between Schwaz and the control districts [9]. In fact, even if the vaccinated adult population of Schwaz may indeed have been less observant of the physical distancing rules after the campaign, we still noted a significant indirect effect on the unvaccinated group of children. 

Lastly, our study had been conducted in the first half of 2021 when the Alpha variant was the predominant variant in Austria and therefore results cannot be directly transferred to later SARS-CoV-2 variants of concern and the Omicron variant. We assume that the level of indirect protection conferred to unvaccinated children by a high vaccine coverage in adults might be similar for the Delta variant as Comirnaty vaccination provides a similar level of protection against acquiring infection with the Alpha and the Delta variant [19]. However, the situation may be different for the Omicron variant as Comirnaty vaccination was less efficient in preventing overall infection with this variant although efficacy against severe COVID-19 disease was still high [20-22]. However, with new, Omicron-specific vaccines rolled out in autumn 2022, our results on indirect protection may become very relevant again.

## Conclusion

Our study provides evidence of an indirect protection effect from a rapid COVID-19 mass vaccination campaign on an unvaccinated group. Given that the vaccination coverage in Schwaz was very similar to the vaccination coverage in many other countries in 2021 (around 70%), our results may also be relevant for other regions and countries.

## Supplementary Data

481000 Supplementary Material
