# Supplementary material for: Can high COVID-19 vaccination rates in adults help protect unvaccinated children? Evidence from a unique mass vaccination campaign, Schwaz/Austria, March 2021
Source: Euro Surveill. 2022 Sep 29;27(39):2101027. doi: 10.2807/1560-7917.ES.2022.27.39.2101027 (PMC9524054; doi:10.2807/1560-7917.ES.2022.27.39.2101027)
Supplement: Supplementary Material [file 2101027_KIMPEL_Supplementary_material.pdf]

Supplementary Appendix

This supplementary material is hosted by *Eurosurveillance* as supporting information alongside the article **'Can high COVID-19 vaccination rates in adults help protect unvaccinated children? Evidence from a unique mass vaccination campaign, Schwaz/Austria, March 2021'**, on behalf of the authors, who remain responsible for the accuracy and appropriateness of the content. The same standards for ethics, copyright, attributions and permissions as for the article apply. Supplements are not edited by *Eurosurveillance* and the journal is not responsible for the maintenance of any links or email addresses provided therein.

Table S1 summarizes the epidemic profiles of Schwaz and the synthetic control. It shows how SARS-CoV-2 infection spread prior to the vaccination campaign along with information on population, geographical area and the number of municipalities within a district. The series on cumulative infections shows the level of infections of up to three weeks (21days) prior the vaccination campaign.

Figure S2 shows off-label vaccination rates of children below 16 in Schwaz and the rest of Tyrol.

Table S1: *Pre-treatment profiles for Schwaz and the synthetic control groups*

**Variable**	**Schwaz**	**Synthetic Schwaz**[1)]
Children below 16 years		
Infections (day -20)	30.78	30.69
Infections (day -14)	284.77	284.64
Infections (day -8)	469.48	469.57
Infections (day -1)	600.32	600.74
Population	12,993	13,336.86
Area (km^2)	1,843	1,491.99
Number of municipalities within district	39	38.28
RMSPE		19.19
Adults between 16 and 50 years		
Infections (day -20)	37.18	36.53
Infections (day -14)	231.06	228.59
Infections (day -8)	422.29	412.01
Infections (day -1)	539.15	554.28
Population	37,652	19,851.01
Area (km^2)	1,843	1,209.55
Number of municipalities within district	39	34.40
RMSPE		18.96

Notes: Infections are measured per 100,000 inhabitants. Days in parentheses indicate the number of infections in the pre-treatment period (e.g., day -20 represents the infections 20 days before the vaccination campaign took place). [1)] For the age cohort of children below 16 years the chosen donors include Hollabrunn (42.0%), Innsbruck-Land (26.7%), Landeck (9.7%), Melk (12.0%), Spittal an der Drau (9.0%) and Zwettl (0.7%). For people between 16 and 50 years the chosen donors are Hartberg-Fürstenfeld (27.2%), Hermagor (6.6%), Reutte (63.5%) and Zwettl (2.7%). The weights for the chosen districts of donor group are reported in parentheses. All other Austrian districts receive zero weight. The RMSPE (Root Mean Squared Prediction Error) measures the difference in infections per 100,000 between Schwaz and the synthetic control group for all pre-treatment periods.

Table S2: *Pre-treatment profiles for Schwaz and the synthetic control groups*

**Variable**	**Treatment Group**	**Control Group**	**p-value**
Number of municipalities	39	10	
Population	2137.1	3431.0	0.158
Population per km^2	148.9	252.2	0.199
Share females (%)	49.3	49.6	0.593
Share age < 16 (%)	18.3	18.2	0.956
Share age > 65 (%)	15.1	15.6	0.575
Migrants per capita	12.7	11.5	0.434
Share secondary education (%)	7.2	9.3	0.107
Share of commuters (%)	71.4	69.3	0.933
Unemployment rate (%)	5.8	5.4	0.334

Notes: Treatment and control group consists of municipalities in Schwaz and the municipalities along the border of the neighbouring districts of Kufstein and Innsbruck-Land. Columns (1) and (2) report mean values, column (3) the p-value of a two-sided t-test with unequal variances on the differences in the respective characteristics.

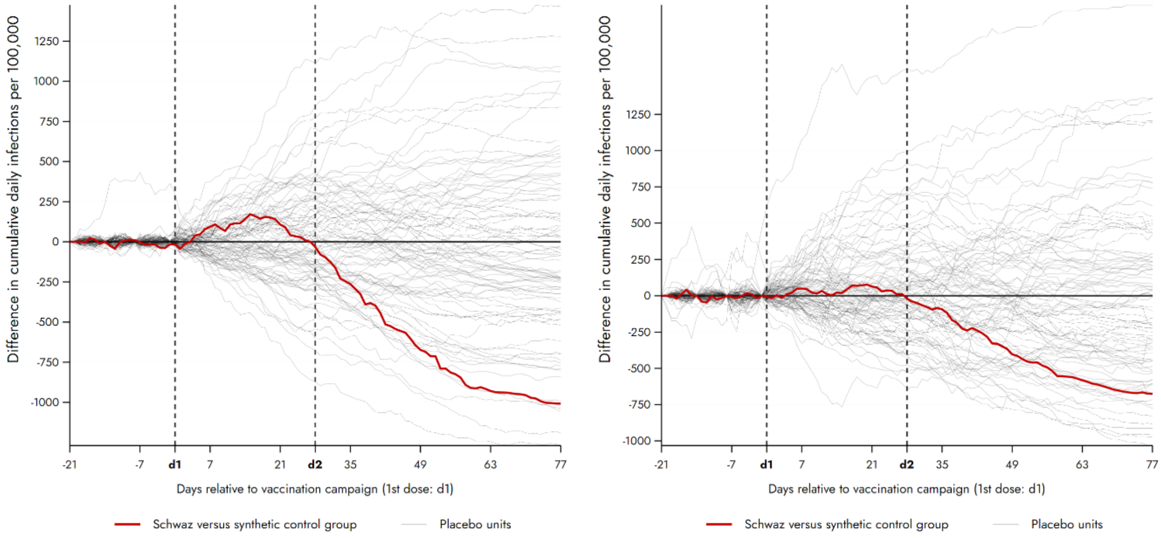

Figure S1. *Placebo-in-space for infections in Schwaz versus all donors*

Notes: Figure S1 describes a placebo test where the SC method is applied to each district in the donor pool (placebo units), using the date of the first dose in Schwaz as the treatment date (11th of March). The figure shows the difference in cumulative daily infections between each of the 91 placebo units and their respective synthetic control units (grey lines), as well as for Schwaz as comparison (red line). A positive (negative) difference indicates higher (lower) cumulative daily infections in the treated group relative to the synthetic control group. Panel a depicts the results for adults between 16 and 50 years, panel b for children below 16 years. The horizontal axis shows the number of days relative to the vaccination campaign (dose 1, indicated by ”d1”). The pre-treatment period started 21 days (three weeks) before the first dose, the post-treatment period ended 77 days (11 weeks) after the first dose. The vertical dashed lines represent the first dose (d1) and the second dose (d2) administered as part of the mass vaccination campaign.

For both age cohorts, the figure shows that the treatment effect for Schwaz was in the upper decile of the placebo distributions. To evaluate the significance of the observed differences between Schwaz and the synthetic control group, we ranked the treatment effects of all 92 districts plotted in Figure 2 in ascending order starting with the highest (negative) effects. Based on this ranking, we employed a Fisher permutation test as proposed by Abadie, Diamond and Hainmueller (2010), leading to a p-value of 0.043 for the results in panel a and 0.096 for the ones in panel b. Based on this, we implemented the procedures described in Firpo and Possebom (2018) to derive confidence sets. In particular, we closely followed the empirical example given in Firpo and Possebom (2018: pp. 20), and chose similar parameters to their application for calculating our confidence sets. Notice that these sets cannot be directly compared with conventional confidence intervals (e.g., the confidence intervals provided for our DID-estimates).

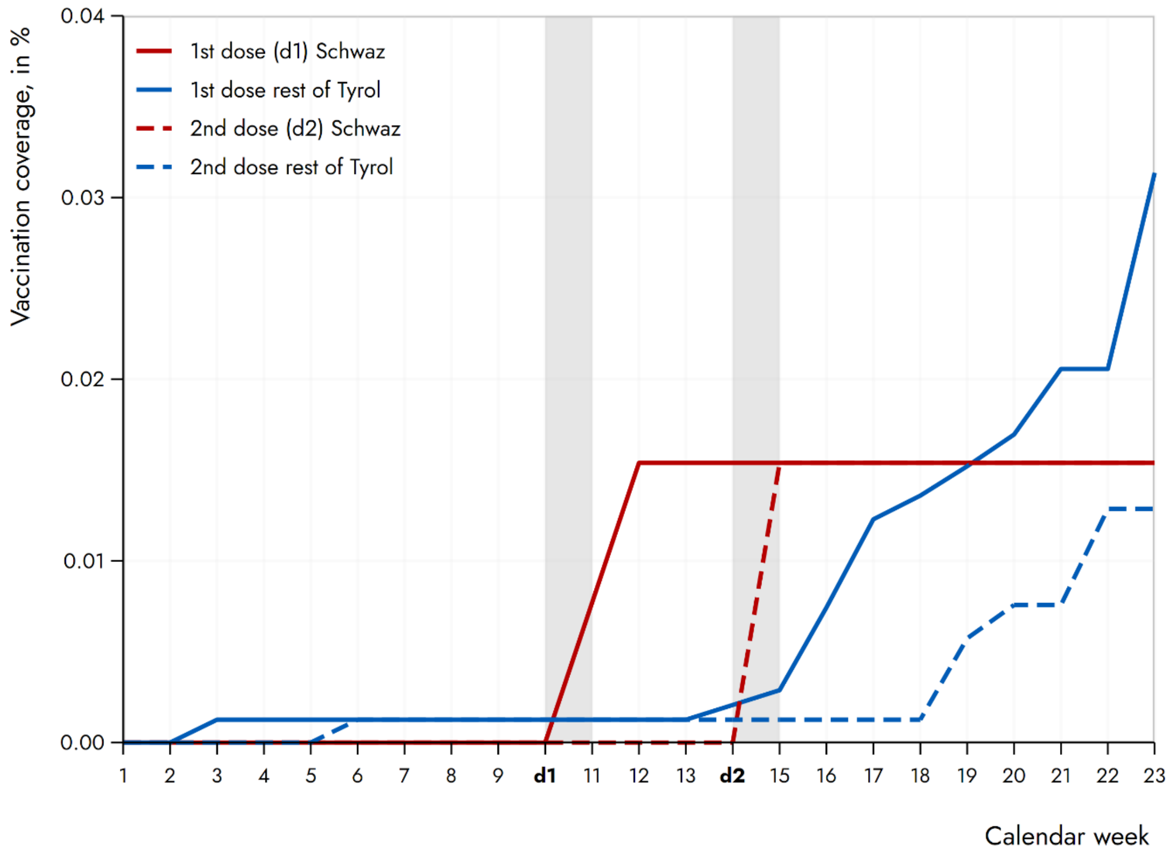

Figure S2. *Vaccination coverage of children below 16 years in Schwaz and the rest of Tyrol*

The figure displays the shares of children below 16 years that received the first (solid line) and second dose (dashed line) of vaccination, respectively. Schwaz is plotted in red, while the other (eight) Tyrolian districts are pooled and depicted in blue. The shaded areas indicate the period of the first (d1: 11th to 16th of March 2021, calendar week 10) and the second (d2: 8th to 11th April 2021, calendar week 14) roll-out of mass vaccination.

References:
Abadie AA, Diamond A, Hainmueller. Synthetic control methods for comparative case studies: Estimating the effect of California's tobacco control program. Journal of the American Statistical Association105:493-505 (2010).

Firpo S, Possebom V. Synthetic Control Method: Inference, Sensitivity Analysis and Confidence Sets. Journal of Causal Inference; 6(2) (2018).
